# Stabilization of Embolized Transcatheter Heart Valve With a Left Subclavian Artery Stent

**DOI:** 10.1016/j.jaccas.2025.104154

**Published:** 2025-07-23

**Authors:** Olufemi T. Olorunda, Ashwat Dhillon

**Affiliations:** Division of Cardiology, Samaritan Health Services, Corvallis, Oregon, USA

**Keywords:** aorta, aortic valve, valve replacement

## Abstract

**Background:**

Transcatheter heart valve (THV) embolization is a rare complication of transcatheter aortic valve replacement (TAVR) that can occur despite meticulous planning and execution.

**Case Summary:**

An 89-year-old man with paradoxical low-flow, low-gradient severe aortic stenosis underwent TAVR. During rapid pacing, a premature ventricular contraction occurred, and the valve embolized to the ascending aorta. A second THV was successfully deployed. The embolized THV was maneuvered into the distal aortic arch, at the ostium of the left subclavian artery (LSCA), and stabilized with an 8 × 39-mm VBX covered balloon expandable stent in the LSCA.

**Discussion:**

Premature ventricular contraction–induced THV embolization is a rare complication of TAVR. We present a novel approach to managing THV embolization in TAVR.

**Take-Home Message:**

If there is jeopardization of the LSCA due to the outer fabric of the THV valve frame, blood flow can be maintained by using a covered stent in the ostial LSCA.

## Visual Summary

**Figure:**
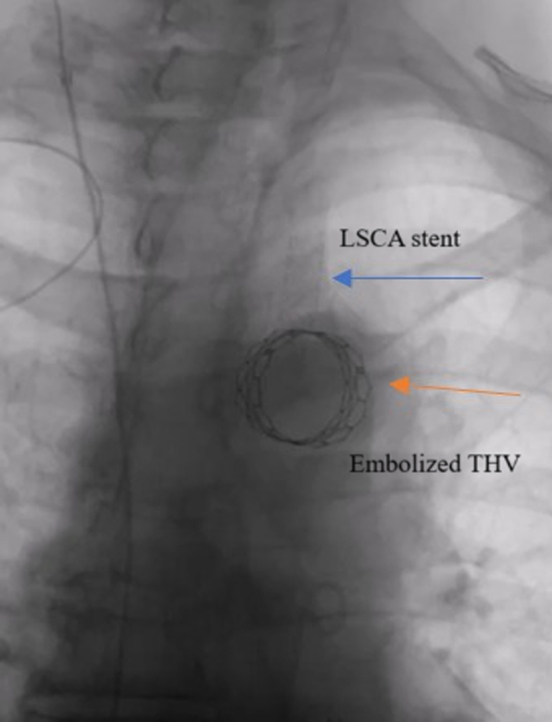

**Visual Summary:**
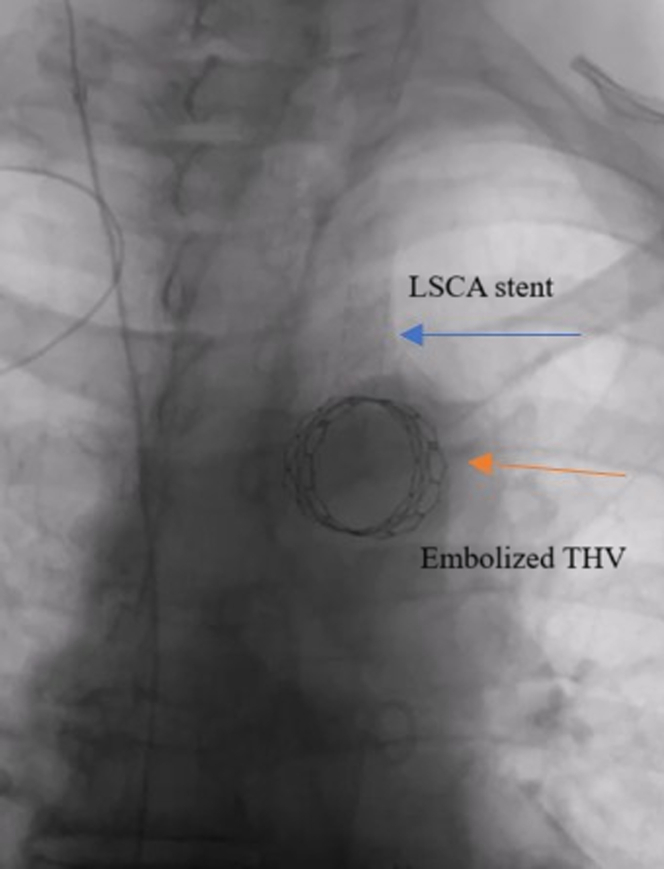
Stabilized Embolized THV With a Covered Balloon Expandable Stent in the LSCA

## Background

Aortic stenosis is present in 5% of the population at 65 years of age, with increasing prevalence with advancing age.^1^ Transcatheter aortic valve replacement (TAVR) is an established treatment modality for all patients with symptomatic severe aortic stenosis.^2^

**Take-Home Message**

• Blood flow can be maintained by using a covered stent in the ostial LSCA, if there is jeopardization of the LSCA due to the outer fabric of an embolized THV frame.

Embolization of the transcatheter heart valve (THV) is a rare complication of TAVR and may occur despite meticulous planning and execution of the procedure.^3^^,^^4^

## History of Presentation

An 89-year-old man with paradoxical low-flow, low-gradient severe aortic stenosis was referred for a TAVR. He endorsed exertional fatigue, dyspnea on exertion, lightheadedness, and diminished exercise capacity. His vital signs were stable. His physical examination was remarkable for a soft S_2_ and a late peaking systolic ejection murmur at the right second intercoastal space.

## Past Medical History

He had a past medical history of colon adenocarcinoma that was resected, hypoparathyroidism, hypertension, and hyperlipidemia.

## Preprocedural Planning

**Figure 1:**
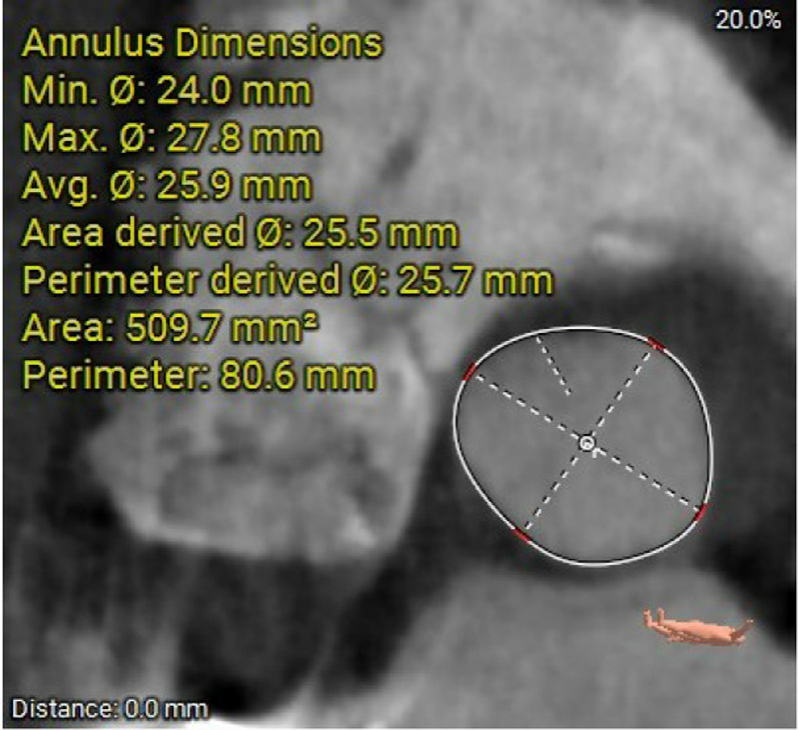
Aortic Annulus Measurements at the 20% Phase of the R-R Cycle

**Figure 2:**
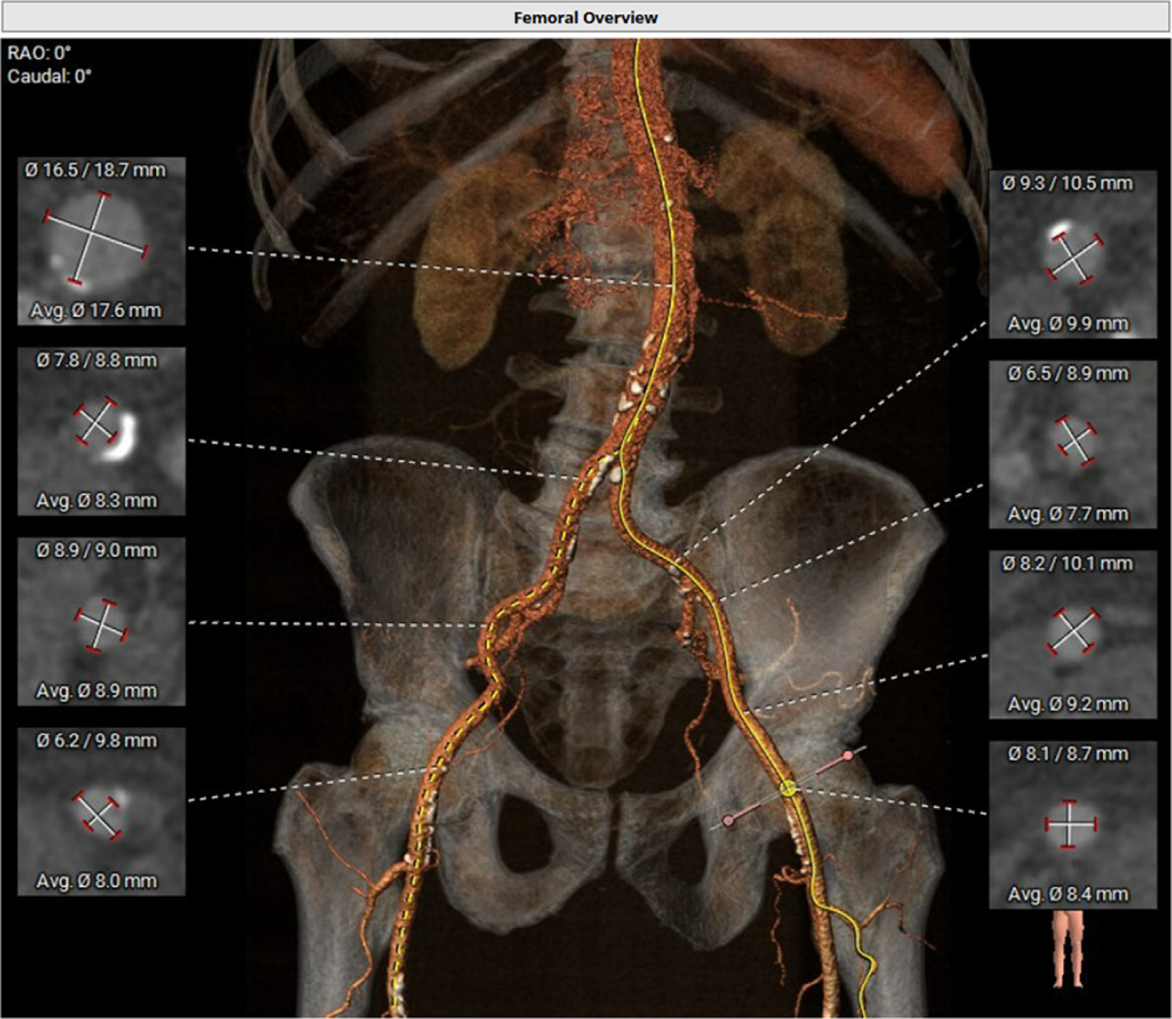
Bilateral Transfemoral Access

Computed tomography angiography with 3-dimensional reconstruction showed the aortic annulus diameter was 24 × 27.8 mm, the annulus perimeter was 80.6 mm, and the annular area was 509.7 mm^2^ at 20% phase of the R-R cardiac cycle (Figure 1). The coronary artery distance to the annular plane was thought to be adequate without risk of obstruction. Iliofemoral anatomy was suitable for transfemoral TAVR (Figure 2). His case was reviewed at multidisciplinary valve conference, and plans were made for a right transfemoral TAVR with a balloon expandable valve (26-mm Edwards SAPIEN 3 Ultra THV).

## Management

Bilateral transfemoral access was obtained and a 6-F sheath was placed in the left femoral artery, a 6-F sheath was placed in the left femoral vein, and a 14-F sheath was placed in the right femoral artery. Aortography was performed confirming views for valve positioning, and the Edwards SAPIEN delivery sheath was placed in the right femoral artery.

The aortic valve was crossed with an 0.035-inch straight wire within an amplatz left 1 catheter. The catheter was exchanged for a pigtail catheter via which a Safari wire was placed in the left ventricle. The 26-mm Edwards SAPIEN 3 Ultra THV was prepped and mounted. The device was placed across the aortic valve, and the position was confirmed with rapid pacing and power injection. During rapid ventricular pacing at a rate of 180 beats/min, the transcatheter valve balloon was manually inflated and deployed.

**Figure 3:**
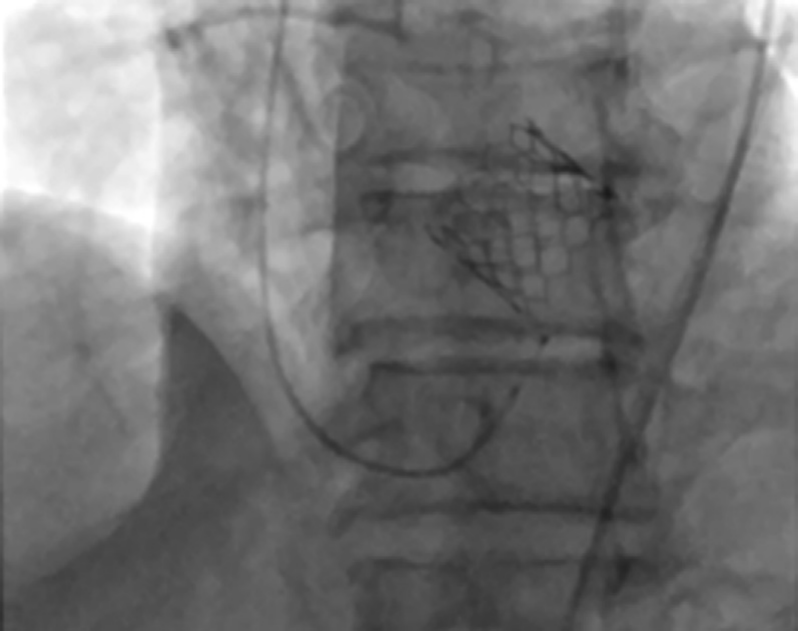
Second 26-mm Edwards SAPIEN 3 Ultra Transcatheter Heart Valve Deployed Successfully in the Aortic Position

During the rapid pacing run for THV deployment, a premature ventricular contraction (PVC) occurred and the valve embolized to the ascending aorta. The embolized THV with the inflated balloon was immediately pulled toward the distal aortic arch, and attempts were made to deploy the THV in the descending thoracic aorta. However, the THV lodged in zone 2 in the aortic arch adjacent to the ostium of the left subclavian artery (LSCA); despite our best efforts using standard techniques and best practices, the embolized THV could not be maneuvered into the descending thoracic aorta. An aortogram was done that showed no dissection and brisk flow in all the great vessels of the aortic arch (Video 1). A second 26-mm Edwards SAPIEN 3 Ultra THV was immediately prepped, mounted, and successfully deployed in the aortic annulus with rapid left ventricular pacing at 180 beats/min without snaring the embolized THV (Figure 3). The embolized THV was not snared while advancing the second THV because of the profile of the balloon expandable valve because there are no tabs, unlike a self-expanding valve that have tabs that facilitate easy snaring.

Intravascular ultrasound of the aortic arch was performed which demonstrated widely patent ostia of the innominate artery and left common carotid artery, but the ostium of the LSCA was partially covered by the fabric skirt of the frame of the embolized THV. An aortogram showed adequate contrast opacification of the LSCA, and an indeterminate haziness in the ostium of the LSCA. Further attempts were made to drag the embolized THV and maneuver it into the descending thoracic aorta, but the attempts were unsuccessful because the embolized THV was larger than the patient's distal aortic arch. Therefore, the decision was made to stent the ostium of the LSCA into the aortic arch through the embolized THV to maintain blood flow in the LSCA, which supplied the dominant left vertebral artery, and to anchor the embolized THV to prevent proximal dislodgement.

**Figure 4:**
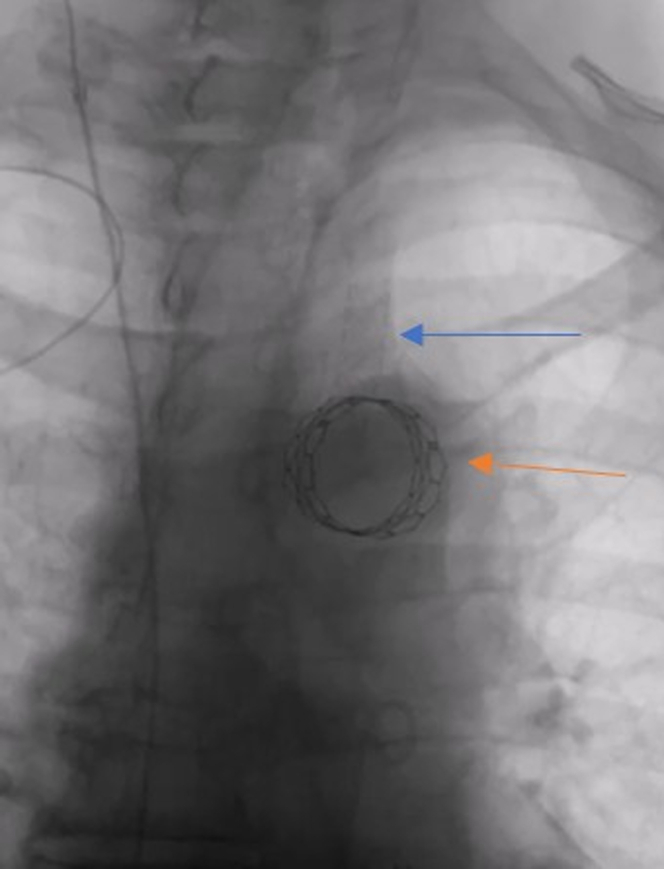
Embolized THV Stabilized With a Covered Balloon Expandable Stent in the LSCA Stabilized embolized transcatheter heart valve (orange arrow) with an 8 × 39-mm VBX covered balloon expandable stent in the left subclavian artery (blue arrow).

A 7-F sheath was placed in the left radial artery. An 0.035-inch wire was manipulated from the LSCA into the descending thoracic aorta (Video 2). An angiogram was performed to mark the left vertebral artery (Video 3). An 8 × 39-mm VBX balloon-expandable covered stent was deployed from the aortic arch to the LSCA (Figure 4, Video 4). Aortogram was repeated which showed brisk antegrade flow into the LSCA with a patent left vertebral artery and brisk flow through the aortic arch into the innominate artery, left common carotid artery, and descending thoracic aorta.

## Outcome and Follow-Up

The patient did fine postprocedure and was discharged home the following day. At his most recent office visit 10 months postprocedure, he had no complaints and was back to his baseline functional status.

## Discussion

The incidence of valve embolization in TAVR has been reported to be 0.1% to 3.7%.^3^^,^^5^, ^6^, ^7^, ^8^ THV embolization typically occurs acutely during the procedure; however, late device migration has also been described postprocedure.^5^^,^^6^ The most common site of THV embolization is the aorta, and less commonly the dislocation occurs caudally into the left ventricle.^5^^,^^9^

The underlying mechanism of embolization in TAVR has been reported to be poor coaxial alignment of the device to the valve plane during implantation, delivery system failure, sizing error, use of self-expanding first-generation and resheathable devices, failure of rapid ventricular pacing, larger aortic root dimensions, complex anatomy, deployment in a high and low position, and eccentric calcification.^5^, ^6^, ^7^ Other causes of embolization include a PVC, poor fluoroscopic angle for implantation, bicuspid aortic valves, postcardiopulmonary resuscitation, embolization during postdilation of the THV, suboptimal anchor, stored wire tension, accidental dislocation during snaring maneuver, manipulation after deployment of the THV, and incomplete or delayed device balloon inflation.^3^^,^^4^^,^^7^^,^^9^^,^^10^

In our patient, a PVC caused embolization of the THV. A second THV was immediately prepped, positioned across the aortic annulus, and deployed successfully. The embolized THV was successfully stabilized in the distal aortic arch and antegrade blood flow maintained in the LSCA by deploying a balloon-expandable covered stent from the aortic arch to the LSCA. This is a useful approach to managing an embolized THV during TAVR, while maintaining blood flow in the aortic arch vessels.

## Conclusions

PVC-induced THV embolization is a rare complication of TAVR. If there is jeopardization of the LSCA due to the outer fabric of the THV valve frame, blood flow can be maintained by using a covered stent in the ostial LSCA, deployed from the left radial artery.

## Funding Support and Author Disclosures

Dr Olorunda owns common stock in Pfizer. Dr Dhillon has reported that he has no relationships relevant to the contents of this paper to disclose.

## Appendix

Video 1 Aortogram Showed No Dissection and Brisk Flow in All the Great Vessels of the Aortic Arch

Video 2 Wire Manipulation From the Left Subclavian Artery Into the Descending Thoracic Aorta Through Embolized Transcatheter Heart Valve

Video 3 Angiogram Performed to Mark the Left Vertebral Artery

Video 4 An 8.0- × 39-mm VBX Balloon-Expandable Covered Stent Deployed From the Aortic Arch to the Left Subclavian Artery
